# Specific Antiproliferative Properties of Proteinaceous Toxin Secretions from the Marine Annelid *Eulalia* sp. onto Ovarian Cancer Cells

**DOI:** 10.3390/md19010031

**Published:** 2021-01-12

**Authors:** Ana P. Rodrigo, Vera M. Mendes, Bruno Manadas, Ana R. Grosso, António P. Alves de Matos, Pedro V. Baptista, Pedro M. Costa, Alexandra R. Fernandes

**Affiliations:** 1 UCIBIO–Applied Molecular Biosciences Unit, Departamento de Ciências da Vida, Faculdade de Ciências e Tecnologia da Universidade Nova de Lisboa, 2829-516 Caparica, Portugal; ar.grosso@fct.unl.pt (A.R.G.); pmvb@fct.unl.pt (P.V.B.); 2 CNC–Center for Neuroscience and Cell Biology, University of Coimbra, 3060-197 Cantanhede, Portugal; vmendes@cnc.uc.pt (V.M.M.); bmanadas@cnc.uc.pt (B.M.); 3 Centro de Investigação Interdisciplinar Egas Moniz (CiiEM), Quinta da Granja, Monte de Caparica, 2829-516 Caparica, Portugal; apamatos@egasmoniz.edu.pt

**Keywords:** invertebrate, cytotoxicity, cancer, ovarian cancer, programmed cell death, cell cycle, toxin

## Abstract

As Yondelis joins the ranks of approved anti-cancer drugs, the benefit from exploring the oceans’ biodiversity becomes clear. From marine toxins, relevant bioproducts can be obtained due to their potential to interfere with specific pathways. We explored the cytotoxicity of toxin-bearing secretions of the polychaete *Eulalia* onto a battery of normal and cancer human cell lines and discovered that the cocktail of proteins is more toxic towards an ovarian cancer cell line (A2780). The secretions’ main proteins were identified by proteomics and transcriptomics: 14-3-3 protein, Hsp70, Rab3, Arylsulfatase B and serine protease, the latter two being known toxins. This mixture of toxins induces cell-cycle arrest at G2/M phase after 3h exposure in A2780 cells and extrinsic programmed cell death. These findings indicate that partial re-activation of the G2/M checkpoint, which is inactivated in many cancer cells, can be partly reversed by the toxic mixture. Protein–protein interaction networks partake in two cytotoxic effects: cell-cycle arrest with a link to RAB3C and RAF1; and lytic activity of arylsulfatases. The discovery of both mechanisms indicates that venomous mixtures may affect proliferating cells in a specific manner, highlighting the cocktails’ potential in the fine-tuning of anti-cancer therapeutics targeting cell cycle and protein homeostasis.

## 1. Introduction

According to the World Health Organization (WHO), cancer is the second leading cause of death globally. The numbers of cases are expected to rise every year despite research on the subject being given top priority (reviewed by Das et al. [1]). From all the types of cancers, breast and lung cancer are the leading causes of death, especially in Europe, with prostate cancer following the rank [2]. Ovarian cancer is the deadliest gynecological cancer in the world (see, for instance, [3]), in large part due to the late diagnosis [4]. This type of cancer is in fact a range of neoplastic diseases that can have different origins [5]. Research on new therapeutics for this disease is motivated by the combed effect between genomic heterogeneity and cell resistance [6]. Moreover, finding a suitable therapy with reduced effects on healthy tissue remains a challenge, leading to the screening for novel anti-cancer compounds that offer more specific and less deleterious strategies than traditional treatments.

Anti-cancer drugs developed from natural products such as toxins and alkaloids nowadays represent 50% of all drugs that are used in chemotherapy [7]. After plants, fungi and bacteria have been leading the search for anti-cancer compounds, in addition to novel antibiotics [7]. Some of the main targets of the quest for anti-cancer drugs are signaling pathways that regulate cell survival and proliferation. Indeed, most research is seemingly focused on the inhibition of specific protein kinases, such as Ras/Raf mitogen-activated protein kinase (MEK) and extracellular signal-regulated kinase (ERK) signaling pathways [8].

There is a growing awareness for the potential of the seas as a source of novel therapeutic agents, in face of their immense biodiversity [7,9]. In fact, the number of marine natural products explored for various biotechnological purposes has been increasing over the past few years (see the review of Hu et al. [10] for a statistical appraisal on marine bioprospecting). Accordingly, there is growing interest in the search for novel anti-cancer compounds in marine life, benefitting from the constant advances in omics methods from metabolomics to proteomics and transcriptomics [11]. With the exception of Yondelis (trabectedin), an approved drug developed from a secondary metabolite (a quinoline) of the tropical tunicate *Ecteinascidia turbinata* that competes with enzyme and transcription factors that bind to DNA [12], most research has yet to be materialized into effect applications. Still, there are promising indications, from various marine animals as varied as the bivalves, the Briozoa and the Porifera, on the effects of novel compounds on the inhibition of DNA synthesis inhibition, RNA polymerase activity, activation of apoptosis and autophagy and cell-cycle arrest in cancer cells [11,13,14,15]. In most cases, though, cancer cells are merely used as a toxicological model, without a clear indication of mode-of-action. From the Polychaeta, a little-explored but most promising group of marine animals for the bioprospecting of novel bioproducts, there are growing indications for the existence of cytotoxic toxins. Among the most significant examples, we may find the toxin arenicin (from *Arenicola marina*), nicomicin (from *Nicomache minor*), both peptidic in nature, and proteases from Nereis, all presenting cytotoxicity towards cancer cell lines, seemingly with pro-apoptotic effects (refer to Rodrigo and Costa [16] and references therein). The mechanisms by which these substances exert noxious effects are not, however, fully understood, if at all. In large part, this is due to constraints in handling the complex mixtures of which poisons, venoms and toxungens are comprised, particularly in marine invertebrates, for whom genomic resources are much reduced.

Recently, we disclosed the secretion of toxins by an intertidal Polychaeta, *Eulalia* sp., whose toxins, secreted by specialized cells in the eversible pharynx, are delivered to its prey using mucus as a vehicle with the purpose of immobilization and cytolysis by means yet unknown [17,18]. In the aftermath of these early findings, the current work aims primarily disclosing the mechanisms by which proteins in *Eulalia*’s secretions become cytotoxic to normal and cancer human cells, with the ultimate goal of asserting their biotechnological potential. To meet our objectives, we isolated proteins from secretions and combined proteomics with transcriptomics for their identification as a means to circumvent the challenges of investigating a complex mixture of proteinaceous compounds and provide inter-validation in a scenario of low genomic resources. The purified extracts were then tested onto both normal and cancer humans cell lines.

## 2. Results

### 2.1. Protein Identification

Five major proteins were conclusively identified in *Eulalia* sp. mucosecretions by superimposing proteomics and transcriptomics (Table 1 and Figure S1 in Supplementary Information). The results identified arylsulfatase B (ARSB), heat shock protein 70 KDa (HSP70), 14-3-3 protein, RAB3 and serine protease (SP) as the main proteins in extracts. When comparing the relative expression of these proteins in the proboscis and body wall, serine protease is the protein with higher expression in the proboscis, followed by ARSB, RAB3 and lastly HSP70 and 14-3-3 protein. The peptide sequences had a 100% match with the translated mRNAs, with the exception of SP, which yielded multiple transcriptional variants (Figure S2).

### 2.2. Cytotoxic Effects

**Figure 1:**
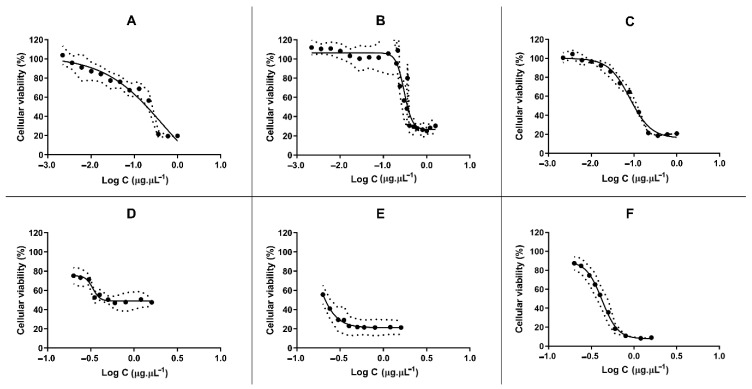
Cell viability determined by the MTS assay in HCT116 (**A**), Fibroblasts (**B**), A2780 (**C**), K562 (**D**), MCF7 (**E**) and A549 (**F**) cell lines. Cells were exposed for 48 h to purified proteins extracts corresponding to concentrations between 0.0022 and 1.6 µg. µL^−1^, plus controls (PBS only). Measurements are indicated as means ± SD of three independent replicates. A positive control (doxorubicin) was used in all assays.

The purified extract caused higher antiproliferative effect towards MCF7 and especially A2780 cancer cell lines, compared to normal human primary fibroblasts (Figure 1, Table 2). Most cancer cells presented a loss of cell viability when exposed to the concentration of the extract within the range of 0.19 to 0.42 µg. µL^−1^, with the exception of the ovarian carcinoma cell line (A2780), which yielded an IC_50_ value one order of magnitude lower (0.08 µg. µL^−1^). Consequently, the ovarian carcinoma cancer cell line was then chosen for further biological characterization towards addressing the mechanisms underlying the loss of A2780 cell viability.

**Figure 2:**
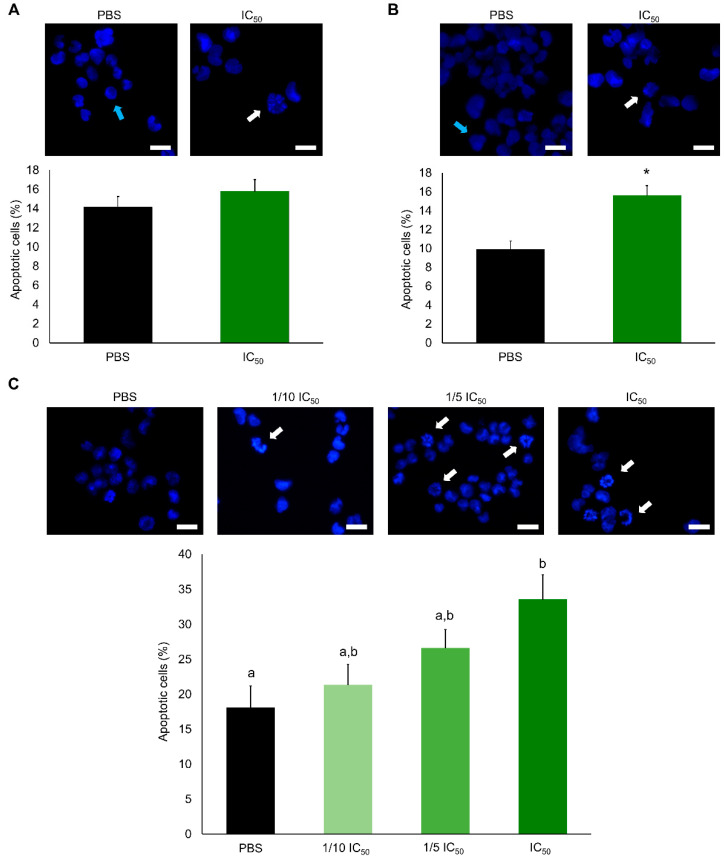
Determination of apoptosis in the A2780 cell line exposed for 12 h (**A**) and 24 h (**B**) to purified extracts corresponding to the IC_50_ threshold (0.08 µg. µL^−1^) and exposed for 48 h (**C**) between 1/10 IC_50_ (0.08 µg. µL^−1^) and IC_50_. Blue arrows are indicative of normal nuclei and white arrows of apoptotic nuclei. Results are presented as mean ± SEM of three independent replicates. *Indicates significant differences towards control (Student’s *t* test, *p* < 0.01). Different letters are indicative of significant differences (Dunn’s test, *p* < 0.05). Scale bars: 10 µm.

**Figure 3:**
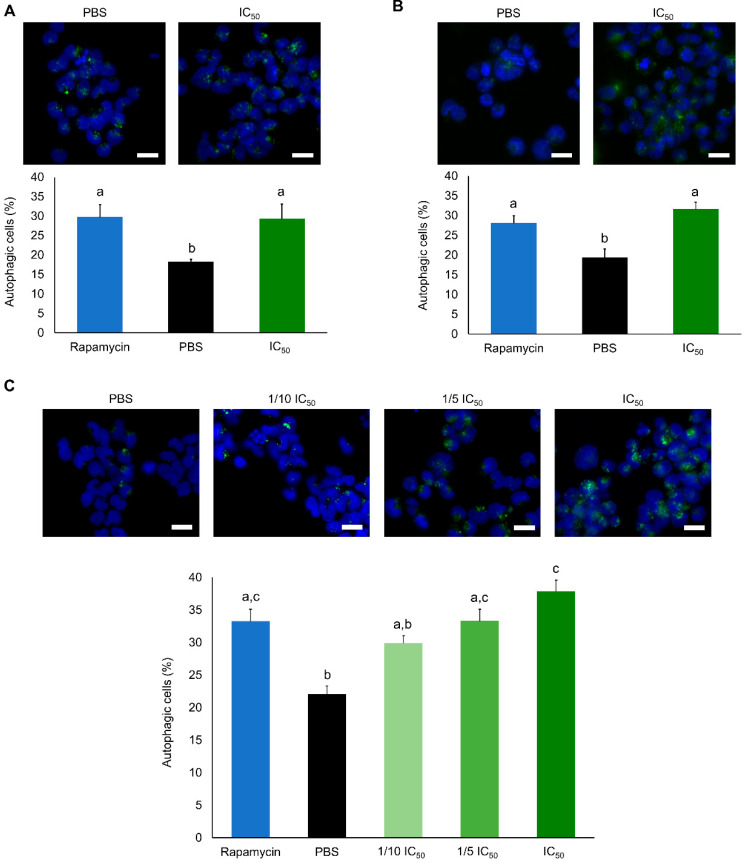
Determination of autophagy in A2780 cell line exposed for 12 h (**A**) and 24 h (**B**) to purified extracts at IC_50_ (0.08 µg. µL^−1^) and exposed for 48 h (**C**) to purified extract between 1/10 IC_50_ (0.08 µg. µL^−1^) and IC_50_. A positive control was used (rapamycin) in all assays. Results are presented as mean ± SEM of three independent replicates. Different letters are indicative of significant differences (Dunn’s test, *p* < 0.05). Scale bars: 10 µm.

**Figure 4:**
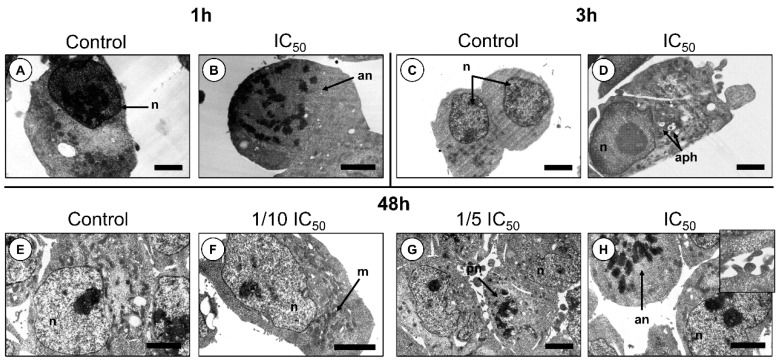
Changes in cell ultrastructure revealed by transmission electron microscopy (TEM) applied to A2780 cells. (**A**) Cells exposed for 1 h to control (PBS). (**B**) Cells exposed for 1 h to IC_50_ concentration. (**C**) Cells exposed for 3 h to control (PBS). (**D**) Cells exposed for 3 h to IC_50_ concentration. (**E**) Cells exposed for 48 h to control (PBS). (**F**) Cells exposed for 48 h to 1/10 IC_50_ concentration. (**G**) Cells exposed for 48 h to 1/5 IC_50_ concentration. (**H**) Cells exposed for 48 h to IC_50_ concentration.. Apoptotic nuclei (an) were visible after 1 h of exposure, whereas autophagosomes (aph) became evident after 3 h. After 48 h of exposure, nuclear pleomorphisms (pn) were more obvious, as well as an increase in the number of mitochondria, regardless of apoptotic or autophagic cells, when compared with controls. Inset: membrane detail of cells exposed to IC_50_ dosage during 48 h. Nuclei (n). Scale bars: 2 µm.

The frequency of apoptotic A2780 cells approximately doubled at IC_50_ concentrations, for both 24 h and 48 h (Figure 2). However, after 12 h, no significant differences were found when the cells were subjected to lower concentrations. Since apoptosis accounted only for a maximum of 35% cell death, an additional mechanism of programmed cell death, namely via autophagy, was assessed (Figure 3). Interestingly, a significant increase in autophagic cell death was observed after 12, 24 and 48 h of exposure. To further confirm the induction of programmed cell death TEM analyses were performed (Figure 4). Apoptotic bodies were easily noticed as early as 1 and 3 h of exposure, as well as chromatin condensation and nuclear fragmentation by blebbing, in accordance with Hoechst labelling (Figure 2). Moreover, autophagolysosomes, associated with the autophagic process, were noticed, especially after 24 h, also supporting the previous fluorescence microscopy results (Figure 3). Exposure to higher concentrations (IC_50_), revealed further cellular alterations, namely mitochondrial shape changes, nuclear pleomorphisms and an increase in lysosome number.

**Figure 5:**
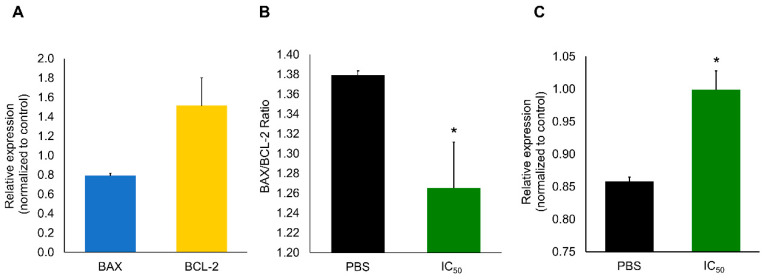
Expression of BAX, BCL-2, their ratio and Caspase 8 activity in A2780 cells exposed to the IC_50_ of purified extracts for 48 h (0.08 µg. µL^−1^). (**A**) Relative expression of BAX and BCL-2, normalized to control. (**B**) Apoptotic index estimated from the BAX/BCL-2 ratio, after incubation with PBS (control) and extract (IC_50_). (**C**) Caspase 8 activity normalized to control. Results are presented as mean ± SEM of three independent replicates. * Indicates significant differences to control (Kruskal–Wallis *H*, *p* < 0.05).

**Figure 6:**
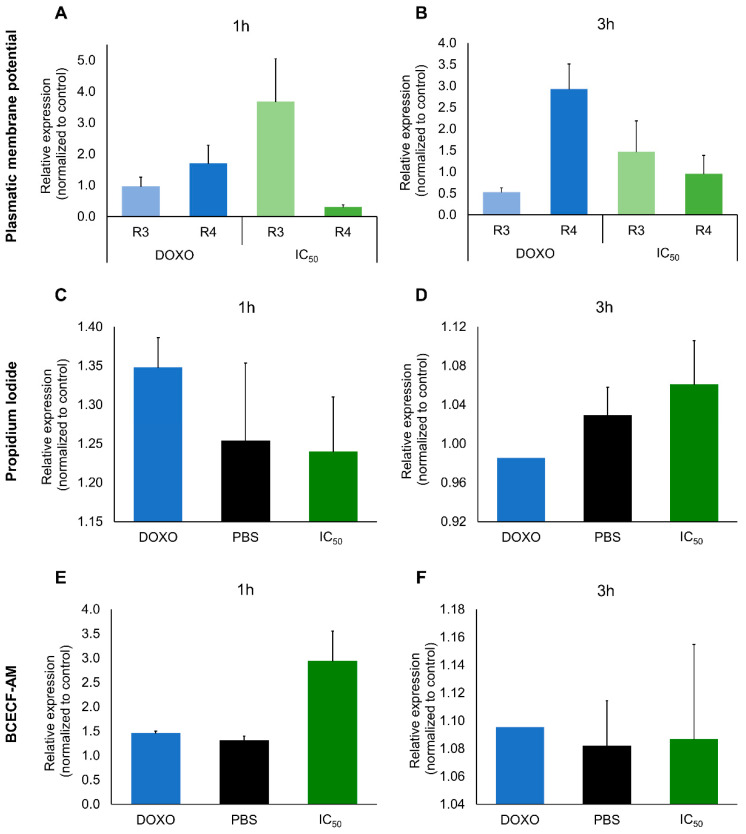
Plasmatic membrane potential (**A**, **B**) and fluorescent staining assays (propidium iodide for nucleic acid staining and BCECF-AM for plasmatic membrane staining, (**C**–**F**) in A2780 cells exposed to IC_50_ extract concentration (0.08 µg. µL^−1^), and the respective control (PBS) for 1 h and 3 h. Results are presented as mean ± SEM of three independent replicates.

For further clarification of the mechanism underlying apoptosis induction, the expression of BAX and BCL-2, the production of ROS, the mitochondrial membrane potential (ΔΨm) and the induction caspase 8 were analyzed in A2780 cells exposed to the IC_50_ of extracts. The low BAX/BCL-2 expression ratio (Figure 5A,B), the absence of ROS production and the unchangeable ΔΨm in A2780 cells exposed to the IC_50_ concentration of the extracts (Figure S2), and the significant induction of caspase 8 activity (Figure 5C) clearly suggests the activation of the extrinsic apoptotic pathway via activation of death receptors. However, alterations to plasma membrane potential (ΔΨp) were noticeable after 1 h and 3 h of exposure, as determined by flow cytometry and fluorescence microscopy (Figure 6A,B).

**Figure 7:**
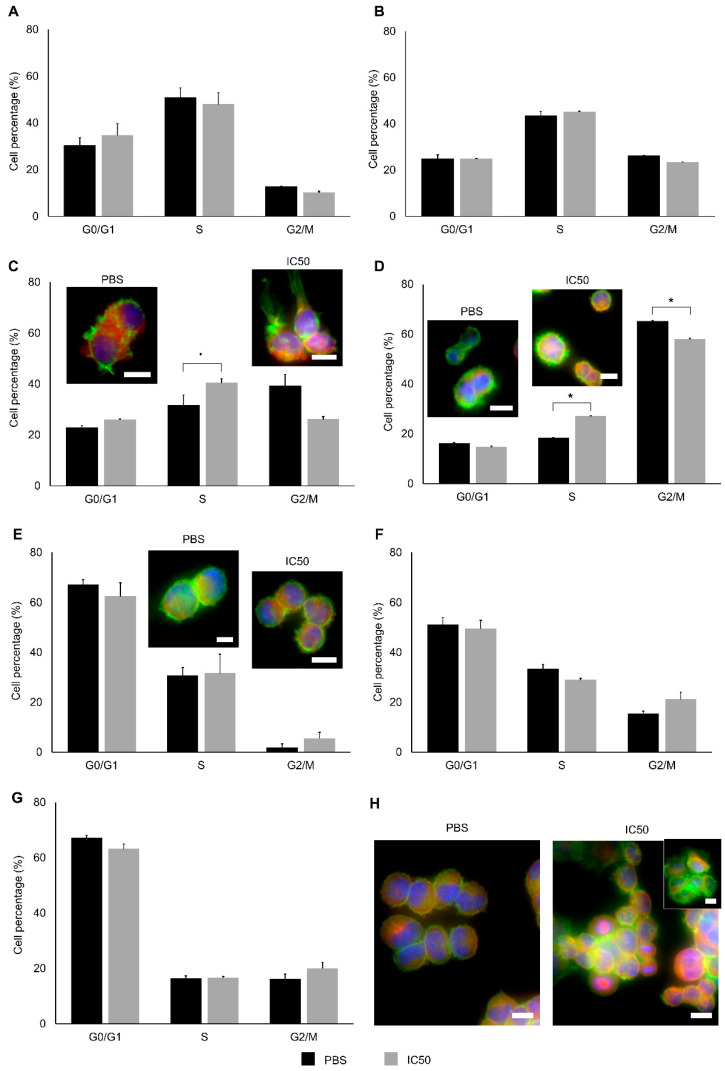
Cell-cycle analysis and immunofluorescence assay at 1 h (**A**), 2 h (**B**), 3 h (**C**), 4 h (**D**), 9 h (**E**), 12 h (**F**), 24 h (**G**) and 48 h (**H**), for A2780 cell line exposed to the extract (IC_50_, 0.08 µg. µL^−1^) and the respective control (PBS). The cell cycle was analyzed by flow cytometry. The cells for the immunofluorescent assay were probed with F-actin (green) and α-tubulin (red) and counterstained with Hoechst dye 33258 (blue). Results are presented as mean ± SEM of three independent replicates. * Indicates significant differences to control (Student’s *t*-test, *p* < 0.05). ▪ Indicates differences to control (*p* < 0.1). Scale bars: 10 µm.

The effect of the extracts in A2780 cell-cycle progression was evaluated over time of exposure (Figure 7). Cell-cycle arrest at phase S was observed 2 h after exposure (Figure 7B), becoming significant after 3 h and 4 h exposure (Figure 7C,D). A slight delay in the percentage of cells entering G2/M phase was also verified after 2 h exposure and extended until 4 h exposure. Alterations in tubulin were also seen with immunohistochemical staining after 3 h exposure. After 4 h exposure, an overcompensation of cells in the phase G2/M started to be noticed, specially at 12 h exposure (Figure 7F). The changes in the early hours were consistent with quantification of both β-actin at 1 h and α-tubulin at 3 h (Figure 6C–F).

### 2.3. Network Analysis

**Figure 8:**
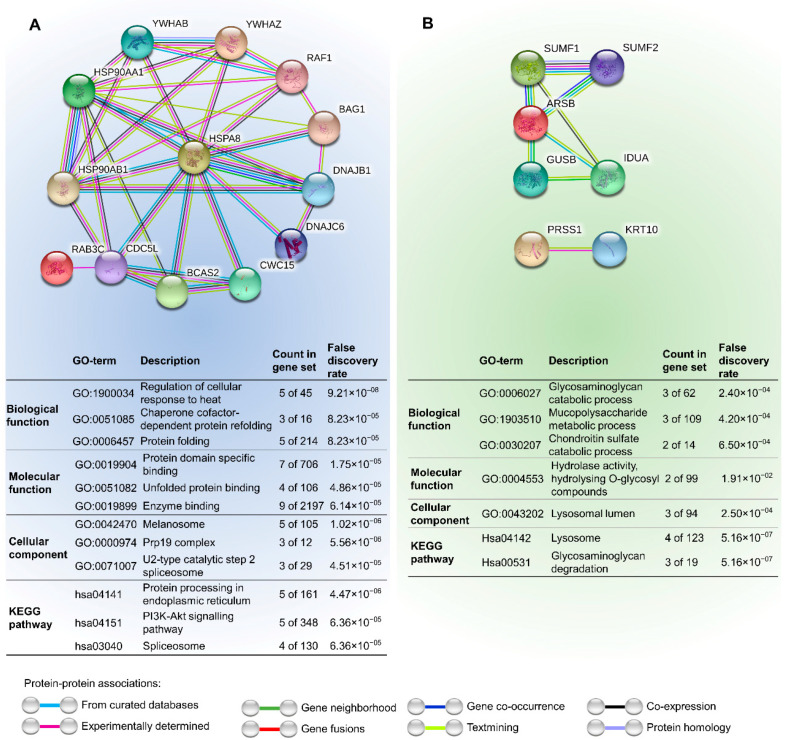
A network analysis between human protein homologs (HSPA8, RAB3C, YWHAZ, ARSB and PRSS1) using STRING. The two autonomous protein interaction networks were expanded to allocate additional ten proteins to increase the radius of interactions beyond the proteins identified in the present work. (**A**) Network with the homologs HSPA8, RAB3C, YWAZ and their respective interactions. (**B**) Network with the homologs ARSB, PRSS1 and their respective interactions. The confidence cut-off for showing interaction links has been set to medium (0.400). ARSB—arylsulfatase B; BAG1—BCL-2 associated athanogene family molecular chaperone regulator 1; BCAS2—Pre-mRNA-splicing factor SPF27; CDC5L—cell division cycle 5-like protein; CWC15—spliceosome-associated protein CWC15 homolog; DNAJB1—DnaJ homolog subfamily B member 1; DNAJC6—putative tyrosine-protein phosphatase auxilin; GUSB—beta-glucuronidase; HSPA8—heat shock cognate 71 kDa protein; HSP90AA1—heat shock protein HSP 90-alpha; HSP90AB1—heat shock protein HSP 90-beta; IDUA—alpha-L-iduronidase; KRT10—keratin, type I cytoskeletal 10; PRSS1—trypsin-1; RAB3C—Ras-related protein Rab-3C; RAF1—RAF proto-oncogene serine/threonine-protein kinase; SUMF1—sulfatase-modifying factor 1; SUMF2—sulfatase-modifying factor 2; YWHAB—14-3-3 protein beta/alpha; YWHAZ—14-3-3 protein zeta.

The interaction between the human homologs of the main proteins present in extracts is depicted in Figure 8. The first network yielded interaction between 14-3-3 zeta protein (YWHAZ), heat shock cognate 71 kDa protein (HSPA8) and Ras-related protein Rab-3C (RAB3C). YWHAZ and HSPA8 yielded the strongest connection with reports of co-expression in humans and other mammals. HSPA8 and RAB3C were also interacting indirectly, trough cell division cycle 5-like protein (CDC5L), a DNA-binding protein involved in cell-cycle control. The connection of HSPA8 and RAB3C and CDC5L have been both supported by experimental data. HSPA8 and CDC5L were also connected with the spliceosome-associated protein CWC15 homolog (CWC15) and Pre-mRNA-splicing factor SPF27 (BCAS2), important proteins that are involved in the spliceosome as well. Due to the lack of direct interactions between PRSS1 and ARSB with the remaining proteins and each other, the radius of the network was expanded to ten proteins. Individually, ARSB is interacting mostly with glycoside hydrolase domain-containing proteins (IDUA, GUSB) and with enzymes responsible for catalyzing the hydrolase of sulphate esters (SUMF1 and 2), while PRSS1 only interacts with keratin (KRT10).

## 3. Discussion

The mucus secreted by the marine worm *Eulalia* contains a mixture of toxin and non-toxin proteins involved in multiple functions for attacking prey and defense against predators and environmental variables (as described in the present work and in Rodrigo et al. [19]). This cocktail of proteins revealed higher toxicity towards the A2780 ovarian cancer cell line, compared to other human cancer cells and even normal fibroblasts, with a strong indication for a dose- and time-dependent responsiveness. We hypothesized that combining proteomics with transcriptomics would shed light on the main proteins involved in cytotoxicity and offer insights on their mode-of-action against A2780 cells, which is seemingly associated to cell-cycle arrest and the induction of programmed cell via extrinsic apoptosis and autophagy. Five proteins were cross-validated by RNA-Seq, whose function, isolated or combined, are responsible for the specific effects in A2780 cells.

The cytolytic properties of animal venoms, poisons and toxungens toward cancer cells has been investigated with promising results, albeit scant comparisons between the differential effects against normal and cancer cells. Bee venom has arguably become one of the most studied and promising cases, with melittin (a 26-amino acid lytic peptide responsible for the painful sting) and phospholipase A being, so far, pinpointed as key substances involved in the promotion of apoptosis [20]. Venoms from snakes also received attention, in research with cancer cell lines inclusively [21,22,23]. For example, crude venom samples from *Naja ashei* induced cell-cycle arrest and promoted apoptosis with the activation of Caspase 3 and 9 in colorectal carcinoma (HCT116) cells [24]. Additionally, venoms from spiders showed high cytotoxic activity towards K^+^ channels expressing cancer cells [25]. Nonetheless, a paramount aspect retrieved from the current findings is the differential cytotoxic effects of the toxin mixture onto different cell cultures, with emphasis on higher sensitivity of A2780 comparatively to normal primary fibroblasts and other cancer cells. The results indicate that the activation of cell proliferation and cell cycle control pathways can be cell type-specific and therefore that the combined effect of toxin proteins act upon such pathways that are more significantly activated in A2780, such as those based on the interplay between BCL-2 and HSP70.

From the five proteins identified from *Eulalia* mucus secretions, arylsulfatase B (ARSB) and serine proteases (SP) are commonly found in venoms [26,27,28]. Sulfatases are known to play a key role in the control of physiological processes like cell signaling, hormone regulation and degradation of complex macromolecules such as glycosaminoglycans [29,30,31]. Arylsulfatase B, specifically, is known to regulate the kininogen–bradykinin axis trough the effect on chondroitin-4-sulfation and the interaction of C4S with kininogen in rat kidney epithelial cells, influencing blood pressure in mammals [32]. In lymphocytes, arylsulfatase may function in the degradation of cerebroside sulphate ester components of the target cell membrane to initiate the lytic event, having an important role in the immune response [33]. In mollusks, it can function as a sulphur-scavenging enzyme, which may play a role in the breakdown of sulphated saponins [28]. The exact function of arylsulfatases in toxic secretions are still unknown, even though this enzyme exists at high quantities in a broad range of organisms, from snakes to predatorial gastropods [27,34]. In turn, serine proteases form one of the largest group of endopeptidases. They are involved in a wide range of physiological processes, from digestion to blood coagulation, fibrinolysis, development, fertilization, apoptosis and play an immunity role as well [35]. In venoms, serine proteases are present in a wide span of animals as well, being well described in snakes and bees. These proteases have thrombin-like effects, promote fibrinogen degradation in prey and activate the phenoloxidase cascade that induces a lethal melanization response in insects attacking bees and their hives [36,37,38].

Other proteins in the toxungenous cocktail of *Eulalia* are not commonly associated with venoms, poisons or toxungens. However, they play paramount roles in cell homeostasis. It is the case of heat shock proteins (HSP). Heat shock protein 70 KDa (HSP70) is usually involved in stress response, helping protein folding (assembly and refolding) in the endoplasmic reticulum [39]. In turn, RAB3C is located in synaptic and secretory vesicles and regulates exocytosis Ca^2+^-dependent secretion and vesicle docking, thus being essential in exocytic and endocytic membrane trafficking [40,41]. The RAB3C protein can also be linked with ZW10-interacting protein 1 (Zwint-1), a protein implicated in the regulation of chromosomal segregation through binding to the kinetochore [42]. The gene encoding this protein, *ZWINT*, was discovered to be overexpressed in ovarian cancer, among other genes (such as *CCNB1*, *CENPF*, *KIF11*) related with cell cycle, nuclear division and oocyte meiosis [43]. The 14-3-3 protein plays a role in various cellular processes like signal transduction, cell-cycle regulation, apoptosis, stress response, cytoskeleton organization and malignant transformation [44]. It must be noted, though, that other proteins may either have not been identified by cross-validation between MS/MS on mucus secretions and skin whole-transcriptome analyses. We must, at this stage, highlight difficulties in homology-based identification of peptides from organisms with reduced genomic annotation. Even though the purification process can, itself, be responsible for the loss of proteins, the toxicological appraisal of the purified extracts does indicate that the active components that yielded effects against A2780 cells were not lost.

The identified proteins, when integrated within protein interaction networks (Figure 8), suggest a potential combined effect. Nonetheless, there are seemingly two pathways: one involving HSPs, 14-3-3 protein and RAB3C, and another where ARSB is pivotal. In the first pathway, 14-3-3 can be the key component, as these proteins are known to play a part in apoptosis and cell-cycle arrest. However, different 14-3-3 isoforms may produce distinct outcomes [44]. Still, the protein here identified holds higher similarities to 14-3-3 protein zeta (YWHAZ), which suggests promotion of programmed cell death. However, some isoforms may have indirect association with BAG1, which may in turn bind to BCL-2 and promote cell survival. It is also able to bind to the serine/threonine-specific protein kinase RAF1 and stimulate RAF1 kinase activity. Moreover, BAG1 also binds to the chaperone HSP70 (HSPA8), being, in fact, a possible chaperone inhibitor [45]. Speculations have been made to whether BCL-2 is able to form channels to transport proteins through membranes and if HSP70 unfolds proteins during such transport [45,46]. In any case, the formation of these channels may contribute to explain BCL-2 overexpression (Figure 5A). At the same time membrane integrity may be compromised by other proteins in the cocktail, explaining the changes in plasmatic membrane potential from 1 h of exposure (recall Figure 6A,B), then likely triggering a series of events that may ultimately lead to cell-cycle arrest and cell death.

The RAF proto-oncogene identified in the network is responsible for the regulation of many fundamental cell processes, including proliferation, differentiation, apoptosis, motility and metabolism [47]. The activation of this protein and the mitogen-activated protein kinase (MAPK) pathway is dependent of proteins present in the network such as HSPA8, HSP90 and CDC protein. These proteins were found to be crucial for the maturation and activation of RAF1 both in *Drosophila* and human cell lines [48,49]. RAF1 activity is also regulated by 14-3-3 proteins, trough phosphorylation, to control its enzymatic activity [50]. The CDC protein is directly connected with CWC15 and BCAS2, both involved in the regulation of gene expression, and is an essential regulator that activates cyclin-dependent kinases (CDKs) at critical stages of the cell cycle [51]. The disruption of the regulatory function can lead to cell-cycle arrest and autophagy, which are the mechanisms by which A2780 cells were affected. More specifically, the 14-3-3 protein present in the extract may well be, directly or indirectly, keeping the cyclin-dependent kinase CDC2 (cell division control protein 2) inactive, thereby preventing G2 to M transition [44,52], as seen in A2780 cells between 2 h to 4 h of exposure (Figure 6). The RAB3C protein is also responsible for cell-cycle arrest through binding to ZW10-interacting protein 1 [42,43]. Additionally, cell-cycle arrest in the S phase is also connected with the depolarization of the resting membrane potential [53], which is in accordance with our results after 3 and 4 h of exposure. After 9 h exposure to the cocktail, cell-cycle progression was promoted when the assays were extended until 48 h. This effect was not, however, sufficient to avoid loss of cell viability.

Heat shock proteins 70 controls many housekeeping processes [54], which is probably the reason why HSPA8 one of their forms, is linked directly with almost all proteins in the network (except RAB3C) described in Figure 8. Two of these interplays occur with other chaperones, namely HSP90 alpha and beta. Besides interfering with cell-cycle progression, HPSs can also be involved in the activation of matrix metalloproteinases. In fact, it has been seen that extracellular HSP90 alpha in fibrosarcoma and breast cancer cells promote MMP2 activation, which is critical for tumor invasiveness [55]. In turn, nuclear HSP70 promotes aurora kinase B (AURKB) activity, which is normally dysregulated in cancer cell lines [56].

Other homologs from known toxins (PRSS1 and ARSB) appeared, at first sight, to have a smaller contribution to the effects seen in A2780. However, ARSB is known to have its expression reduced in most carcinomas when compared with normal tissues [57]. However, as the authors refer, there are some cases of cancer cells where arylsulfatases (ARS) are overexpressed (ARSA and ARSB), as in human lung cancer, pertaining to one of the cell lines tested that yielded lower toxicity of extracts, comparatively to fibroblasts. The overall reduction in the expression of ARS was found to be related to prostate cancer aggressiveness, due to the association between ARSB and the epidermal growth factor receptor (EGFR), commonly overexpressed when ARSB is reduced [58,59]. The EGFR has a crucial role in cell differentiation and proliferation and cancer, being ARSB regulation an important target for cancer therapy [59].

Finally, it must be noted that the co-occurrence of apoptosis and cell death by autophagy has already been reported in A2780 cells exposed, e.g., to metal-based compounds [1,60,61]. Both pathways, occurring simultaneously, were also reported in a cervical cancer cell line, where the accumulation of autophagic vacuoles preceded the apoptotic cell death [62]. However, all the studies have been performed with isolated compounds, while the present study dealt with a mixture of proteins, leading to complex interactions and likely intricate downstream effects on cell homeostasis. Even though the combined effect of toxins secreted by marine invertebrates has received little attention, there are indications that venomous mixtures are more effective against human cancer cells than individualized components, such as in a recent study with glycoconjugates extracted from a starfish tested on colorectal carcinoma cells [63].

## 4. Materials and Methods

### 4.1. Protein Extraction and Identification

#### 4.1.1. Animal Collection

Adult *Eulalia* sp. (≈120 mm total length and weighting ≈250 mg each) were collected from the West Coast of Portugal (38°41′42″ N, 09°21′36″ W), in an intertidal rocky beach. Animals were reared in the laboratory in a mesocosm environment recreating their natural habitat, with controlled salinity, temperature and photoperiod (35 ± 1, 16 ± 1 °C and 10:16 h, respectively). Animals were fed with live mussels, one of the species’ favorite prey.

#### 4.1.2. Protein Collection and Purification

Protein was collected from mucous secretions harvested non-invasively by placing the worms in microfuge tubes with filtered sterile seawater, followed by mechanical stimulation of the proboscis, leading to increased secretion. Crude protein extracts were obtained from approximately fifty animals per pool. The crude extracts were immediately diluted ≈ 1:1 in 0.05 M Tris-HCl containing 10% (*w*/*v*) L-dithiothreitol (DTT) and 1% (*v*/*v*) protease inhibitor cocktail (Sigma-Aldrich, St. Louis, MO, USA). Samples were then filtered through a syringe cellulose acetate filter (0.22 µm). Purification and concentration of extracts was done by dialysis (ultrafiltration) using 3kDa Amicon Ultra centrifugal filters (Merck, Algés, Portugal), with Dulbecco’s phosphate-buffered saline (DPBS, pH 7.4) as the vehicle. Total protein content was determined using a Nanodrop 1000 spectrophotometer (Thermo Fisher Scientific, Waltham, MA, USA). Purified extracts were stored at −80 °C until further analyses.

#### 4.1.3. Protein Separation

Proteomics was employed to analyze the purified protein extracts using either, one- or two-dimensional gel electrophoresis (1DE and 2DE, respectively) for initial separation [64]. The Laemmli discontinuous gel of Laemmli [65] was used for 1DE separation by sodium dodecyl-sulphate polyacrylamide gel electrophoresis (SDS-PAGE), as detailed by Hames [66]. Stacking and running gels (8 × 9 cm by 0.75 mm thick) contained 12% (*v*/*v*) and 6% (*v*/*v*) acrylamide, respectively. The migration buffer consisted of 25 mM Tris, 192 mM glycine, pH 8.5. Molecular standards (NZYColour Protein Marker II range 11-245 kDa) was purchased from Nzytech. The 2-D separation was obtained by isoelectric focusing (IEF) followed by SDS-PAGE. Briefly, the sample consisted of 100 μL of purified protein extract treated with 2-D Clean-Up Kit (GE Healthcare, Chicago, IL, USA), according to manufacturer’s instructions. Precipitated proteins were resuspended in 100 μL of rehydration solution containing 4% (*w*/*v*) CHAPS, 7 M urea, 2 M thiourea, 1% (*w*/*v*) DTT GE Healthcare, 0.5% (*v*/*v*) Immobilized pH Gradient buffer (GE Healthcare) and 0.002% (*w*/*v*) bromophenol blue, and incubated at room temperature for 36 h. After protein quantification using the Pierce 660 nm protein assay reagent (Thermo Fisher Scientific, Waltham, MA, USA), 125 μL of rehydration buffer containing 200 μg of protein were applied onto Immobiline Drystrip IPG strips (GE Healthcare) with 7 cm and 3 ± 10 non-linear pH. After rehydration, Isoelectric focusing (IEF) was done in an Ettan IPGphor instrument (GE Healthcare) at a maximum voltage of 5000 V. The IPG strips were afterwards incubated for 15 min in equilibration buffer 1 (50 mM Tris-HCl pH 8.8, 2% (*w*/*v*) SDS, 6 M urea, 30% (*v*/*v*) glycerol, 1% (*w*/*v*) DTT, followed by incubation for another 15 min in equilibration buffer 2 (50 mM Tris-HCl pH 8.8, 2% (*w*/*v*) SDS, 6 M urea, 30% (*v*/*v*) glycerol, 2.5% (*w*/*v*) iodoacetamide). Strips were then placed on top of a 10% SDS-PAGE gel for separation by mass. Both gels were stained with Coomassie Brilliant Blue G-250 to identify most representative spots and bands (Figure S1) to be picked for liquid chromatography coupled to tandem mass spectrometry (LC-MS/MS) analyses. Excised gel bands and spots were stored at −80 °C until further processing.

#### 4.1.4. Protein Identification by LC-MS/MS

After gel sample destaining and digestion with porcine trypsin, peptides were analyzed on a Eksigent NanoLC Ultra 2D separation system coupled to a TripleTOF 6600 mass spectrometer, all from Sciex (Framingham, MA, USA). The chromatographic separation by micro-LC was achieved in a ChromXP C18CL column (0.3 × 150 mm, 3 μm, 120 Å, from Eksigent), at 50 °C. The flow rate was set to 5 μL/min and mobile phase A and B were 0.1% (*v*/*v*) formic acid plus 5% (*v*/*v*) DMSO in water and 0.1% (*v*/*v*) formic acid plus 5% (*v*/*v*) DMSO in acetonitrile, respectively. The ESI DuoSprayTM ionization source was operated in the positive mode set to an ion spray voltage of 5500 V, 25 psi for nebulizer gas 1 (GS1), 25 psi for the curtain gas (CUR). The rolling collision was used with a collision energy spread of 5. Peptide mass fingerprinting was performed using ProteinPilot 5.0.1 (Sciex) considering the following parameters: cysteine alkylation by acrylamide, digestion by trypsin, and gel-based ID as a special factor. The resulting amino acid sequences were contrasted against UniProtKB and NCBInr databases using Blast [67]. Accuracy of identification was determined by the lowest *e*-values and number of matching peptides per protein.

#### 4.1.5. Protein Identification by RNA-Seq

The peptide sequences were contrasted against *Eulalia* sp. proboscis transcriptome [19], deposited in Gene Expression Omnibus (GEO) under the accession number GSE143954 (Figure S2). The results from RNASeq were validated by RT-qPCR. In brief: cDNA was synthesized from the total RNA samples using the First-Strand cDNA Synthesis Kit (NZYTech). Primers were designed using Primer Blast and verified in silico with Oligo Analyzer (Table S1) to amplify an expressed sequence tag (EST) for mRNAs coding for proteins identified by MS/MS and RNA-Seq. The calibrator was GAPDH, as suggested [68]. Amplification was done in a Biometra Gradient Thermocycler96. Following resolving PCR products in an agarose gel, these were Sanger-sequenced (as a service), translated and matched against (1) UNIPROT cluster UniRef90 [69] by generating a customized toxin database, with BLASTP v2.5.0 [70], having set a maximum *e*-value of 10^−5^; (2) NCBInr using BlastP. The RT-qPCR was then done in a Rotor-Gene 6000 thermal cycler (Corbett Research) using the NZY qPCR Green Master Mix (NZYTech), according to manufacturer instructions. The program was set as: denaturation 94 °C, 45 s (s), annealing 55 °C, 25 s and extension 72 °C, 30 s, with 45 cycles per run. Primer melting analysis was also conducted to verify the specificity of hybridization. Relative expression was determined by the 2^−∆∆Ct^ method [71].

### 4.2. Network Analysis

After the identification of the main proteins by combining proteomics and transcriptomics, *Homo sapiens* homologues were retrieved using BLASTP. Gene network analyses were conducted using the Search Tool for the Retrieval of Interacting Genes/Proteins (STRING) by setting the confidence cut-off for interaction links between proteins at 0.400 [72].

### 4.3. Cell-Based Assays

HCT116 (human colorectal carcinoma), A549 (lung adenocarcinoma), human primary fibroblasts, MCF7 (breast carcinoma) and K562 (myelogenic leukemic carcinoma) cells were grown in Dulbecco’s modified Eagle’s medium (DMEM) (Invitrogen, Grand Island, NY, USA) supplemented with 10% (*v*/*v*) fetal bovine serum and 1% (*v*/*v*) antibiotic/antimycotic solution (Invitrogen), while A2780 (ovarian carcinoma) cells were grown in modified McCoy’s medium: Roswell Park Memorial Institute medium (RPMI) 1630 [73,74]. All cell lines were purchased from ATCC (http://www.atcc.org) with the exception of A2780 that was purchased from Sigma-Aldrich. All cell cultures were maintained at 37 °C in a humidified atmosphere containing 5% (*v*/*v*) CO_2_ and incubated in the same atmosphere when necessary. Only purified extracts stored for less than 30 days (−80 °C) were used in biological assays and all dilutions (and controls) were prepared in PBS. The same volume of PBS was added to the respective medium of each assay (controls). Total protein content was determined prior to biological assays using a Nanodrop spectrophotometer (Thermo Fisher Scientific). The assays were conducted with cells seeded for 24 h and in the range of 10^5^ cells. mL^−1^, in the conditions described before, in 6, 24 or 96-well plates, depending on the endpoint.

#### 4.3.1. Cell Viability

Cell viability was determined by subjecting all cell lineages to successive dilutions of the purified protein extract (2 × 10^-3^ to 1.6 µg. µL^−1^) for the estimation of the half-maximal inhibitory concentration (IC_50_). Cells were incubated for 48 h with each diluted extract. Viability was determined with the CellTiter 96 AQueous Non-Radioactive Cell Proliferation Assay (Promega, Madison, WI, USA), using 3-(4,5-dimethylthiazol-2-yl)-5-(3-carboxymethoxyphenyl)-2-(4-sulfophenyl)-2Htetrazolium, inner salt (MTS). Quantification of the formazan dye formed in viable cells was done spectrophotometrically by measuring absorbance at 490 nm with a Bio-Rad microplate reader Model 680 (Bio-Rad, Hercules, CA, USA) [73,74,75]. The assays were performed in triplicate and technical duplicates were included, to minimize technical error. Doxorubicin (0.4 mM) was used as a positive control in all assays.

#### 4.3.2. Apoptosis

Apoptosis was determined based on the Hoechst 33258 labelling assay. Cells were seeded on sterilized coverslips and incubated for 24 h to obtain an adherent monolayer. Cells were then incubated with the purified extract for 12 h (IC_50_), 24 h (IC_50_) and 48 h (1/10 IC_50_, 1/5 IC_50_ and IC_50_). After incubation, cells were washed with PBS and fixed with fresh 4% (m/v) formaldehyde (reconstituted from paraformaldehyde), for 10 min in the dark. Cells were then incubated with the dye (provided by Sigma-Aldrich), prepared as 5 μg. mL^−1^ in PBS. After 10 min, slides were washed with PBS and mounted. Fluorescent nuclei (100 per sample) were sorted in a DM 2500 LED model microscope, adapted for epifluorescence with an external EL6000 light source (Leica Microsystems, Wetzlar, Germany). The assay was performed in triplicate for each timeline. The results are expressed as the mean percentage of apoptotic nuclei.

#### 4.3.3. Autophagy

Cells were exposed to the extracts for 12 h, 24 h and 48 h, at the same concentrations than the previous assay. Rapamycin (50 mM) was used as a positive control [61,74]. By the end of each assay, the medium was removed, and cells were stained with the CYTO-ID Autophagy Detection Kit (ENZO, New York, NY, USA), following manufacturer instructions. Stained cells were imaged with the aforementioned epifluorescence microscope following counterstaining with DAPI. The assay was performed in triplicate for each timeline.

#### 4.3.4. Caspase 8 Assay

The activity of Caspase 8 was determined to assess the extrinsic apoptotic pathway using the Caspase 8 assay kit from Abcam, following manufacturer instructions. Briefly, cells were plated on T-flasks with an area of 25 cm^2^ with 6 × 10^5^ cells. mL^−1^ for 24 h and exposed to the purified extract (IC_50_ dose), plus PBS (control) and incubated for 48 h. Cells were then scraped, washed in PBS, resuspended in cell lysis buffer and incubated on ice for 30 min. Total protein in lysates was quantified as previous and adjusted to 200 µg/50 µL buffer. Each microplate well was loaded with 200 µg protein/50 µL 50 µL of 2× Reaction Buffer with DTT (100 mM final concentration) and 5 µL of IETD-pNA substrate (4 M). After incubation (2 h), absorption at 400 nm was quantified on a 680 Microplate Reader (Bio-Rad, Hercules, CA, USA).

#### 4.3.5. Detection of Reactive Oxygen Species

Intracellular reactive oxygen species (ROS) were determined using the KITNAME (Sigma-Aldrich), as previously described [1]. Briefly, cells were seeded and incubated for 24 h, after which the culture medium was removed and replaced by fresh medium containing 1/10 IC_50_, 1/5 IC_50_ and IC_50_ (0.08 µg. µL^−1^), plus controls. Hydrogen peroxide (50 µM) was used as a positive control. Cells were harvested after 48 h, washed twice with PBS before resuspension in pre-warmed (37 °C) PBS containing 100 µM 2,7-dichlorodihydrofluorescein diacetate (H_2_DCF-DA) and incubation at 37 °C for 20 min, in the dark. Fluorescence intensity was measured on an Eclipse Ti inverted microscope equipped with a DS-QiMc camera and adapted for epifluorescence (all from Nikon), with fixed exposure time for all samples. The green fluorescent signal was normalized and analyzed using ImageJ [76].

#### 4.3.6. Mitochondrial Transmembrane Potential

The mitochondrial transmembrane potential was analyzed using the bis(5,6-dichloro-1,3-diethyl-2-benzimidazole)trimethinecyanine iodide (JC-1) dye (Abnova Corporation, Walnut, CA, USA). After seeding and incubation cells were treated with the protein extract at the concentrations of 1/10 IC_50_, 1/5 IC_50_ and IC_50_ diluted in fresh medium and exposed for 48 h. Cells were then stained with the JC-1 staining solution for 20 min at 37 °C in the dark. Image acquisition and analysis was done as above, based the ration between green and red fluorescence.

#### 4.3.7. Plasmatic Transmembrane Potential

The plasmatic transmembrane potential was analyzed using 2′,7′-Bis(2-carboxyethyl)-5(6)-carboxyfluorescein tetrakis(acetoxymethyl) ester (BCECF-AM) dye (Invitrogen). After seeding and incubation, cells were treated with the purified protein extract at the concentration of IC_50_, diluted in fresh medium (RPMI), and incubated for 1 h and 3 h. Cells were then trypsinized and washed with PBS and RPMI medium (without phenol red) before incubation with BCECF-AM (2 µM in the same medium) for 20 min, at 37 °C. After washing in the culture medium, data was collected using an Attune acoustic focusing flow cytometer (ThermoFisher Scientific) and analyzed using FCS Express 6.0 Flow Cytometry (De Novo Software, Pasadena, CA, USA). The results were normalized for cell counts and the respective control. The assays were performed in duplicate and technical duplicates were included to minimize technical error. Doxorubicin (0.4 mM) was used as a positive control in all assays.

#### 4.3.8. Interference of Cellular pH or Membrane Integrity

After seeding and incubation, cells were treated with the protein extract at the concentration of IC_50_ diluted in fresh RPMI medium for 1 and 3 h. After washing with RPMI (without phenol red), cells were incubated with BCECF-AM (µM) for 20 min, washed again and incubated with propidium iodide (2.5 µg. mL^−1^) for 10 min. Cells were then washed and visualized in RPMI (without phenol red) under an Eclipse Ti fluorescence microscope, equipped with a DS-QiMc camera (Nikon Instruments, Amsterdam, Netherlands).

#### 4.3.9. Western-Blot

Cells were plated on T-flasks with an area of 75 cm^2^ with 4 × 10^5^ cells. mL^−1^. After incubation for 24 h to allow adhesion, cells were treated with fresh medium supplemented with purified extract at the concentration of IC_50_ or PBS (control) and incubated for 48 h. The cell monolayer was washed three times with PBS and the cells were detached by scraping. The cells were pelletized by centrifugation at 500× *g* for 5 min (4 °C). The following procedure was adapted from Vinhas et al. [77], using a PVDF membrane (GE Healthcare). Signal acquisition was obtained with Hyperfilm ECL (GE Healthcare). The protein band intensities relative to controls was quantified using ImageJ [78] following normalization to β-actin values.

#### 4.3.10. Cell-Cycle Analysis

Cells were seeded in a 6-well plate at 1 × 10^5^ cells per well and synchronized in early S-phase using a thymidine double block [74]. After the second block, the medium was replaced with fresh medium containing the extract or control (PBS only). The cells were collected by trypsinization after 1 h, 2 h, 3 h, 4 h, 9 h and 24 h. After centrifugation at 650× *g* for 5 min at 4 °C, pelleted cells were washed with 1 mL cold PBS and centrifuged at 3000× *g* for 5 min at 4 °C. The cells were then suspended in 100 µL cold PBS and completely disaggregated by soft pipetting before gently adding 1 mL ice-cold ethanol (80% (*v*/*v*)). After at least 16 h at 4 °C, the cells were pelletized by centrifuging at 5000× *g* for 10 min at 4 °C and the supernatant discarded. The cells were treated for 30 min with RNase A 50 µg. mL^−1^ at 37 °C and propidium iodide was added to a final concentration of 25 µg. mL^−1^. Data was collected using an Attune acoustic focusing cytometer (ThermoFisher Scientific) and analyzed using FCS Express 6 Flow Cytometry (De Novo Software).

#### 4.3.11. Determination of Cytoskeletal Alterations

Cells were seeded for 24 h in 24-well plates at 1 × 10^5^ cells. mL^−1^ for 3 h, 6 h and 12 h and at 0.375 × 10^5^ cells. mL^−1^ for 48 h. Cells were then fixed in 4% (*v*/*v*) formaldehyde, permeabilized with 0.1% (*v*/*v*) Triton X-100 and blocked with 1% (*w*/*v*) bovine serum albumin (BSA). The cells were probed with Alexa Fluor 488 Phalloidin (2.4% (*v*/*v*) in 1% (*w*/*v*) BSA, 20 min) to stain actin filaments, followed by Hoechst 33258 (Sigma, Missouri, USA) at 5 μg. mL^−1^ in PBS (15 min) as the nuclear counterstain. Alpha tubulin was tagged with the DM1A anti-α-tubulin monoclonal antibody (Sigma-Aldrich), diluted to 1:200 in 1% (*w*/*v*) BSA. After incubation o/n at 4 °C, the primary antibody was tagged with anti-mouse TRITC-conjugated polyclonal secondary antibody (Sigma), diluted to 1:64 in 1% (*w*/*v*) BSA (1 h). Cells were then washed with Tris-buffered saline (pH 7.6) with Tween 20 (TBST) and mounted with PBS. The cells were then examined with an Eclipse Ti fluorescence microscope (Nikon Instruments), as described previously.

#### 4.3.12. Cytology

Cells, seeded in 24-well plates with a density of 10^6^ cells. mL^−1^, were exposed to different concentrations of the purified extract (IC_50_, 1/5 IC_50_, 1/10 IC_50_), plus controls (PBS), for 48 h and additionally to IC_50_ for 1 h and 3 h. After exposure, cells were scraped and processed for transmission electron microscopy (TEM) as described in Vogt et al. [79], with modifications. Briefly, cells were fixed in 2.5% (*v*/*v*) glutaraldehyde in 0.1 M phosphate buffer and post-fixed in 2% (*w*/*v*) osmium tetroxide in 0.1M phosphate buffer, overnight. Pellets were washed in ultrapure water and dehydrated in a progressive series of acetone. Pelletized cells were embedded in Epon resin (Sigma-Aldrich) using propylene oxide as intermediate. Thin sections (50–60 nm) were obtained with an Ultracut E ultramicrotome Leica. Sections were collected onto copper and nickel mesh grids and contrasted with 2% (*w*/*v*) aqueous Uranyl Acetate and Reynold’s Lead Citrate [80]. Grids were analyzed using a JEOL 100-SX model TEM operated at 80 kV.

#### 4.3.13. Statistical Analysis

The half maximum inhibitory concentration (IC_50_) was estimated using least squares regression and the results are expressed as IC_50_ ± standard error of estimates (SEE). Normality and homoscedasticity were evaluated using Shapiro-Wilk’s and Levene’s tests, respectively. When assumptions were met for parametric analyses, we employed ANOVA based on *F* and Dunn’s tests for multiple comparisons, otherwise the non-parametric Kruskal–Wallis *H* statistic was used, followed by Student’s *T*-test and Mann-Whitney’s *U* (parametric and non-parametric tests, respectively) for paired-sample tests. Statistics were computed using R 3.5 [81]. The significant level was set at 0.05 for all the analyses.

## 5. Conclusions

In large part due to the number and variety of compounds therein, toxin-bearing secretions of marine invertebrates may have cytotoxic properties beyond the functions acquired by means of natural selection. The present study showed that proteins present in the mucus secreted by a toxungenous marine worm, five of which were positively identified, can specifically cause cell-cycle arrest by a mechanism that is specific to A2780 ovarian adenocarcinoma cells among a panel of six human cell lines, normal included. Even though the precise mechanisms still need further exploration, the effects on A2780 cells seem to derive from the collision of two interaction networks: one centered in cell-cycle arrest as a consequence of dysregulation of protein expression and folding, with a link to RAB3C oncoprotein function; another centered in the lytic activity of arylsulfatases, common enzymes in animal venoms and poisons. The mechanisms, however, are complex and the exact quantities of each individual component in the extract may vary. Nevertheless, the variation in this case seems to be neglectable, as the usage of biological replicates gave similar results. Thus, the consistency of the findings supports the potential of mixed proteins in venoms against aggressively-proliferating cells, as seen, e.g., for bee venoms, steering toward a novel approach to anti-cancer treatments while highlighting the immense potential of the oceans as a source of novel therapeutics.

## Figures and Tables

**Table 1:** Matched peptidic sequences and translated mRNAs upregulated in the proboscis and respective contrasting against Pfam and Toxins databases.

Protein	Method	logFC	Conserved Domain (Pfam)	Protein Match	e-Value (Blastp)
SP	RNAseq	11.85	Trypsin-like serine protease	Serine protease 33-like (*Orbicella faveolata*)	6 × 10^−54^
	MS/MS		-	Serine protease (*Protobothrops flavoviridis*)	6 × 10^−5^
	MS/MS		-	Transmembrane protease serine 7 (*Ophiophagus hannah*)	7.8
ARSB	RNAseq	5.08	ALP P like superfamily	Arylsulfatase B (*Penaeus vannamei*)	2 × 10^−83^
	MS/MS		-	N-acetylgalactosamine 6-sulfate sulfatase (*Candidatus Moranbacteria bacterium*)	19
RAB3	RNAseq	3.96	Rab GTPase family 3	Rab3 (*Doryteuthis pealeii*)	2.00 × 10^−147^
	MS/MS		Rab GTPase family 3	Ras family protein (*Trichuris suis*)	1.00 × 10^−06^
	MS/MS		-	Ras-related protein Rab-3 (*Melipona quadrifasciata*)	0.45
14-3-3	RNAseq	1.53	14-3-3 superfamily	14-3-3 zeta (*Pristionchus pacificus*) (38kDa)	2.00 × 10^−121^
	MS/MS		-	14-3-3-like protein 4 (*Pseudotsuga menziesii*)	0.21
HSP70	RNAseq	1.09	Heat shock 70 kDa protein	HSPA8 protein (*Homo sapiens*)	0
	MS/MS		HSP70	Heat shock cognate 71 kDa protein (*Cricetulus griseus*)	3.00 × 10^−15^

**Table 2:** Half maximal inhibitory concentration (IC_50_) determined through the cell viability assay (MTS assay). Values for the IC_50_ determined for each cell line are indicated as means ± SEE of three independent replicates.

Cells	IC_50_ ± SEE (µg. µL^−1^)
A2780	0.08 ± 0.01
HCT116	0.24 ± 4.00
A549	0.46 ± 0.05
MCF7	0.15 ± 0.04
K562	0.37 ± 0.04
Fibroblasts	0.29 ± 0.02

## Data Availability

Bulk data is available as Supplementary Information. Bulk RNA-Seq data is deposited in Gene Expression Omnibus (GEO) under the accession number GSE143954.

## Supplementary Materials

The following are available online at https://www.mdpi.com/1660-3397/19/1/31/s1, Figure S1. Image of proteins identified by MS/MS; Figure S2. Alignment of the peptides obtained from MS/MS and RNAseq; Table S1. Sequences of primers used in RT-qPCR; Figure S3. Expression analysis of key proteins in the mucus extract by RT-qPCR, comparing the proboscis and body wall; Figure S4. Production of Reactive Oxygen Species (A) and Mitochondrial Membrane Potential (B) in A2780 cells exposed during 48 h to the extract (IC_50_, 0.08 µg. µL^−1^) and the control (PBS); Figure S5. Autophagy assay of cells exposed to the extract and the control (PBS) during 12 h, 24 h and 48 h to an extract concentration of IC_50_ (0.08 µg. µL^−1^) and during 48 h to a concentration of 1/10 IC_50_ and 1/5 IC_50_; Figure S6. Immunofluorescent assay used in A2780 cells exposed to the extract (IC_50_, 0.08 µg.µL^−1^) and the control (PBS) during 3 h, 6 h, 12 h and 48 h; Table S2. Individual results from STRING for each of the homologous in *Homo sapiens* (ARSB-arylsulfatase B, PRSS1-trypsin-1 serine protease, YWHAZ-14-3-3 protein zeta, RAB3C-Ras-related protein Rab-3C, HSPA8-Heat shock protein 70 kDa), for the proteins identified in *Eulalia* sp.
