# Supplementary material for: Specific Antiproliferative Properties of Proteinaceous Toxin Secretions from the Marine Annelid Eulalia sp. onto Ovarian Cancer Cells
Source: Mar Drugs. 2021 Jan 12;19(1):31. doi: 10.3390/md19010031 (PMC7827603; doi:10.3390/md19010031)
Supplement: Supplementary file 1 [file marinedrugs-19-00031-s001.pdf]

Specific Antiproliferative Properties of Proteinaceous Toxin Secretions from the Marine Annelid *Eulalia* sp. onto Ovarian Cancer Cells

Ana P. Rodrigo [1,*], Vera M. Mendes [2], Bruno Manadas [2], Ana R. Grosso [1], António P. Alves de Matos [3], Pedro V. Baptista [1], Pedro M. Costa [1,*] and Alexandra R. Fernandes [1,*]

[1] UCIBIO – Applied Molecular Biosciences Unit, Departamento de Ciências da Vida, Faculdade de Ciências e Tecnologia da Universidade Nova de Lisboa, 2829-516 Caparica, Portugal; ar.grosso@fct.unl.pt (A.R.G.); pmvb@fct.unl.pt (P.V.B.)

[2] CNC – Center for Neuroscience and Cell Biology, University of Coimbra, 3060-197 Cantanhede, Portugal; vmendes@cnc.uc.pt (V.M.M.); bmanadas@cnc.uc.pt (B.M.)

[3] Centro de Investigação Interdisciplinar Egas Moniz (CiiEM), Quinta da Granja, Monte de Caparica, Caparica, Portugal; apamatos@egasmoniz.edu.pt (A.P.A.)

* Correspondence: a.rodrigo@campus.fct.unl.pt (A.P.R.); pmcosta@fct.unl.pt (P.M.C.); ma.fernandes@fct.unl.pt (A.R.F.); Tel.: +351-212-948-300

This PDF file includes:

Figures S1 to S6

Tables S1 to S2

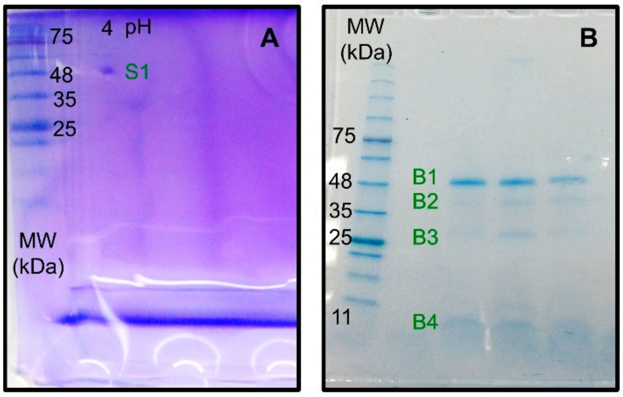

Figure S1. Image of proteins identified by MS/MS. **A**) Spot obtained from 2-DE gel (S1 – arylsulfatase B). **B**) Bands retrieved from SDS-PAGE gel (B1 – heat shock protein 70, B2 – 14-3-3 protein, B3 – rab3, B4 – serine protease).

ARSB

```
    MS  --------------------------------------------------------------------------------
RNAseq  MRLLFAIGLLAWTANGQGGGGPGGGGPSGMDTSSIMSSLADQTGGLGTSTRPSCNLHRGTSQPNIVFFLADDLGWNDV
    MS  --------------------------------------------------------------------------------
RNAseq  SWHNPDVVSPRMQELAETGVILERQYSQPVCSPSRGTLFTGKFAHRLGMQHNVLGAATPECLHLSERIISNSLKRLGY
    MS  --------------------------------------------------------------------------------
RNAseq  TNHYLGKWHLGYCDEECTPLARDFDSFYGFFNGFVDHYTHEFAGNFDWRDGTEAGFSVKGEYATDLITTRAVDIINSH
    MS  --------------------------------------------------------------------------------
RNAseq  DGSTPLFMFVSQAAVHTPIQTPPDVEASDDVRTNYLTMLTAMDTSIGTIVDAMKDAGLYDNSIIIFASDNGGENGPSS
    MS  --------ATLFEGGTR---------------------------------------------------------------
RNAseq  NHPLRGEKATLFEGGTRSASFVHSPLLENPGRIADGMEGTFHFADWHPTIYAMAGGCESNGMANMDGMDLSGMVMRGE
                ********* 

    MS  --------------------------------------------------------------------------------
RNAseq  PSSRTEFPYQLDQYQPAPFGHAAIRVGEYKLIWGYPGLFDGWEGDADYPVGQTQIVDVFGLGATGGGRKKRGDYSFDW
    MS  --------------------------------------------------------------------------------
RNAseq  GAIMEYEAKCANHTWLYNVIEDPEERTDISAGNERIVAAMKNRIVSMFSTDVVDIDLASLADSPEAYNVLDEDPDTGD
    MS  ----------
RNAseq  LYWAPGWCDF*
```

HSP70

```
    MS  --------------------------------------------------------------------------------
RNAseq  QDVLLLDVTPLSRGIEPAGGVMTVLIKRNTTIPTKQTQTFTTYSDNQPGVLIQVYEGERAMTKDNNLLGKFELTGIPP
    MS  ---GVPQIEVTFDIDANGILNVSAVDK-----------------------------------------------------
RNAseq  APRGVPQIEVTFDIDANGILNVSAVDKSTGKENKITITNDKGRLSKEDIERMVQEAEKYKAEDEKQRDKVSSKNSLES
           ************************

    MS  --------------------------------------------------------------------------------
RNAseq  YAFNMKATVEDEKLQGKINDEDKQKILDKCNEIINWLDKNQTAEKEEFEHQQKELEKVCNPIITKLYQSAGGMPGGMP
    MS  ------------------------
RNAseq  GGFPGGGAPPSGGASSGPTIEEVD*
```

14-3-3 zeta

```
    MS  --------------------------------------------------------------------------------
RNAseq  MSDQDSAAREELVQYAKLAEQAERYDDMADKMKKVVTKMSTELSNEERNLLSVAYKNVVGARRSSWRVLSSIEQKDKS
    MS  --------------------------------------------------------------------------------
RNAseq  AEKQAFTKDYRRQVEQELTKICKEVLDLLDQYLIKEVGPPESKVFYLKMKGDYYRYLAEVESNDEDKRAGKSSEVVEC
    MS  ----------------------------------------------------------------------DSTLIMQLLR
RNAseq  SEKAYKEALAVAEKELAPTHPIRLGLALNFSVFYYEITNAPDNACKLAKQAFDDAIAELDTLKEDSYKDSTLIMQLLR
                                                                              **********

    MS  --------------------------
RNAseq  DNLTLWTSDQTDDDEQPQQPSGGGDE*
```

Rab 3

```
    MS  -----------------------LLIIGNSSVGK----------------------------------------------
RNAseq  MAAAGDSKWQKDAADQNFDYMFKLLIIGNSSVGKTSFLFRYADDSFTSAFVSTVGIDFKVKTVFRQDKRVKLQIWDTA
                               ***********

    MS  ------------------------------------------TYSWDNAQVVLVGNK-----------------------
RNAseq  GQERYRTITTAYYRGAMGFILMYDITNEESFNAVQDWCTQIKTYSWDNAQVVLVGNKCDLEEERVVSTERGKQLADQL
                                                  ***************

    MS  ----------------------------------------------------------------------------
RNAseq  GLLFFETSAKENQCLEFFETSAKENINVKAVFERLVDIICDKMSESLDSDPTIVNNQTGGKRLTAEANPQNGGCQC
```

Serine protease

```
    MS  --------------------------------------------------------------------------------
RNAseq  MSGLMFLVVFGTLVVTVSAKSTFGGHVHRSESKMREALEQLSKDLKTETYHCGQPVSSSRIVGGHQSEANQWPWMLRL
    MS  --------------------------------------------------------------------------------
RNAseq  NLDGDLYCGASILSRNWALTAAHCLYEDPMGAGGGYNYGYFGNGGNDVGGDGNNNNYYWYWYSLRGRGSGATRITDPS
    MS  --------------------------------NDDEVDKDIMLIK-----------------------------------
RNAseq  RLSVTAADHNIQQTESQEQTVQVQVIYEHPRFDLDSVENDLALLKLTTPLAFNDKVRQVCLPTADPPAGTECVATGWG
                                        : *.*::*: *:*

    MS  --------------------------------------------------------------------------------
RNAseq  ATSDAGASPSTLNQVTLPLYSRPACAAAIEGLADNQFCAGRPQGGVDTCQGDSGGPLVCPINGYWVQVGVTSFGDECA
    MS  ----PGVYTK--------------------
RNAseq  APNSPGVYTRLSKYQEWIAQTTGNAA-DFS*
            *****:
```

Figure S2. Alignment of the peptides obtained from MS/MS and RNAseq. Similarities are indicated as follow: "*" - Indicative of fully conserved residue, ":" – Indicative of strongly similar properties – scoring > 0.5 in the Gonnet PAM 250 matrix, "." – Indicative of weakly similar properties – scoring ≤ 0.5 in the Gonnet PAM 250 matrix.

Table S1. Sequences of primers used in RT-qPCR. Primers were designed using primer blast (www.ncbi.nlm.nih.gov/tools/primer-blast/index.cgi, National Center for Biotechnology Information) and their quality assessed using OligoAnalyzer Tool (www.idtdna.com/pages/tools/oligoanalyzer, Integrated DA Technologies) and optimized by PCR.

Target	Forward primer (5'-3')	Tm (°C)	Reverse primer (5'-3')	Tm (°C)	Amplicon
Gap_dh	CAGGGGTGCTAAGCAGT TGG	60.96	GAGAAGGCTGGGGCTCA TTT	60.03	165
Rab3	CAGTTGCTCTCATCCAC GGT	60.04	AGCCTGGAATGGAAGTC ACG	60.04	136
14-3-3	GTCCGTTGTTTGACACG CTC	60.04	GACCCACAACTCCTACT GGC	60.04	155
ArsB	GACACTGGCGACCTCTACTG	59.83	GGTAGCTCTCCTTTGTGC GT	60.04	155
SP	ACGGGGTTGCTTAATTCG	59.96	AGCCACACTAATGCGAA CGA	60.04	158
Hsp70	TCCCGTACTCTTGTCCACAG	58.75	AGCGTGCCATGACAAAG GAT	60.32	152

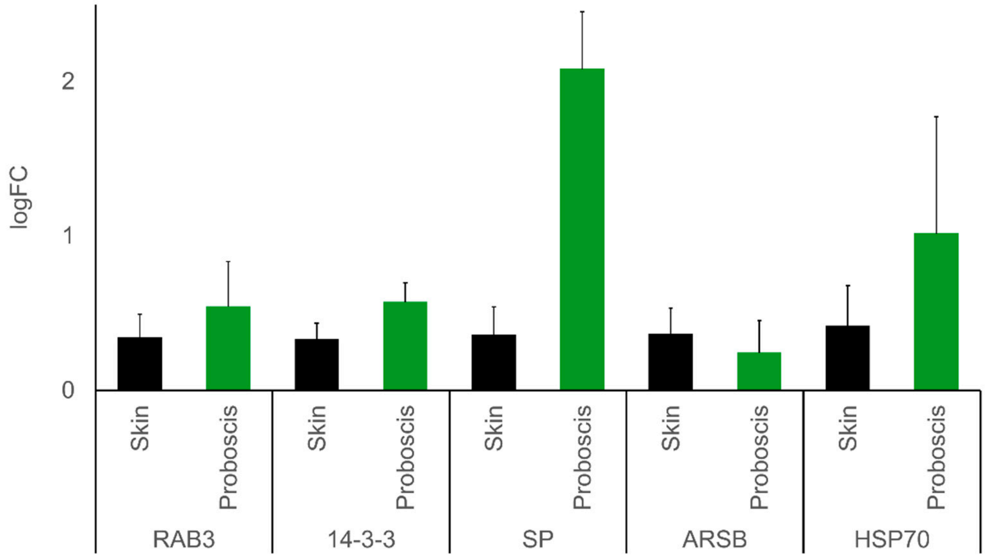

Figure S3. Expression analysis of key proteins in the mucus extract by RT-qPCR, comparing the proboscis and body wall. Data are expressed as mean relative expression of *Rab3*, *14-3-3* protein, *ArsB* (Arylsulfatase B), *SP* (Serine protease) and *Hsp70* (Heat-shock protein 70 kDa). The housekeeping gene Gap-dh was used as calibrator.

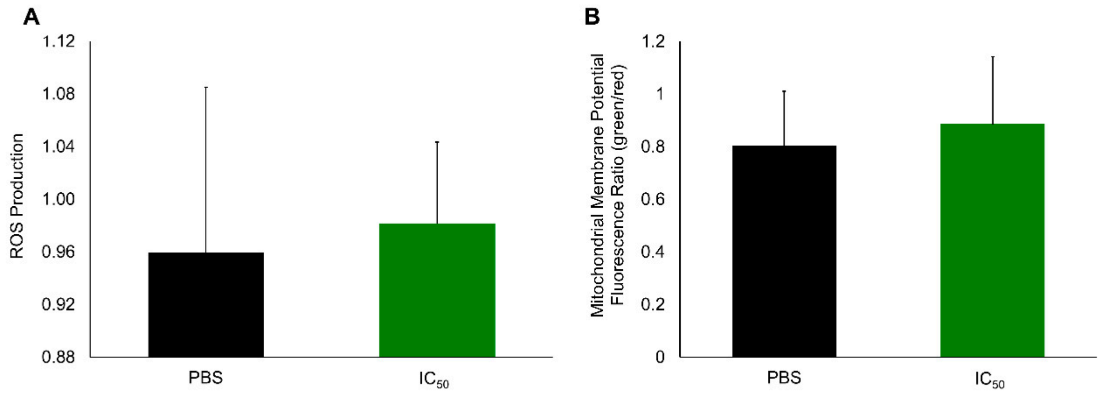

Figure S4. Production of Reactive Oxygen Species (**A**) and Mitochondrial Membrane Potential (**B**) in A2780 cells exposed during 48 hours to the extract (IC_{50}, 0.08 μg. μL^{-1}) and the control (PBS). No significant statistical differences were found between treatments.

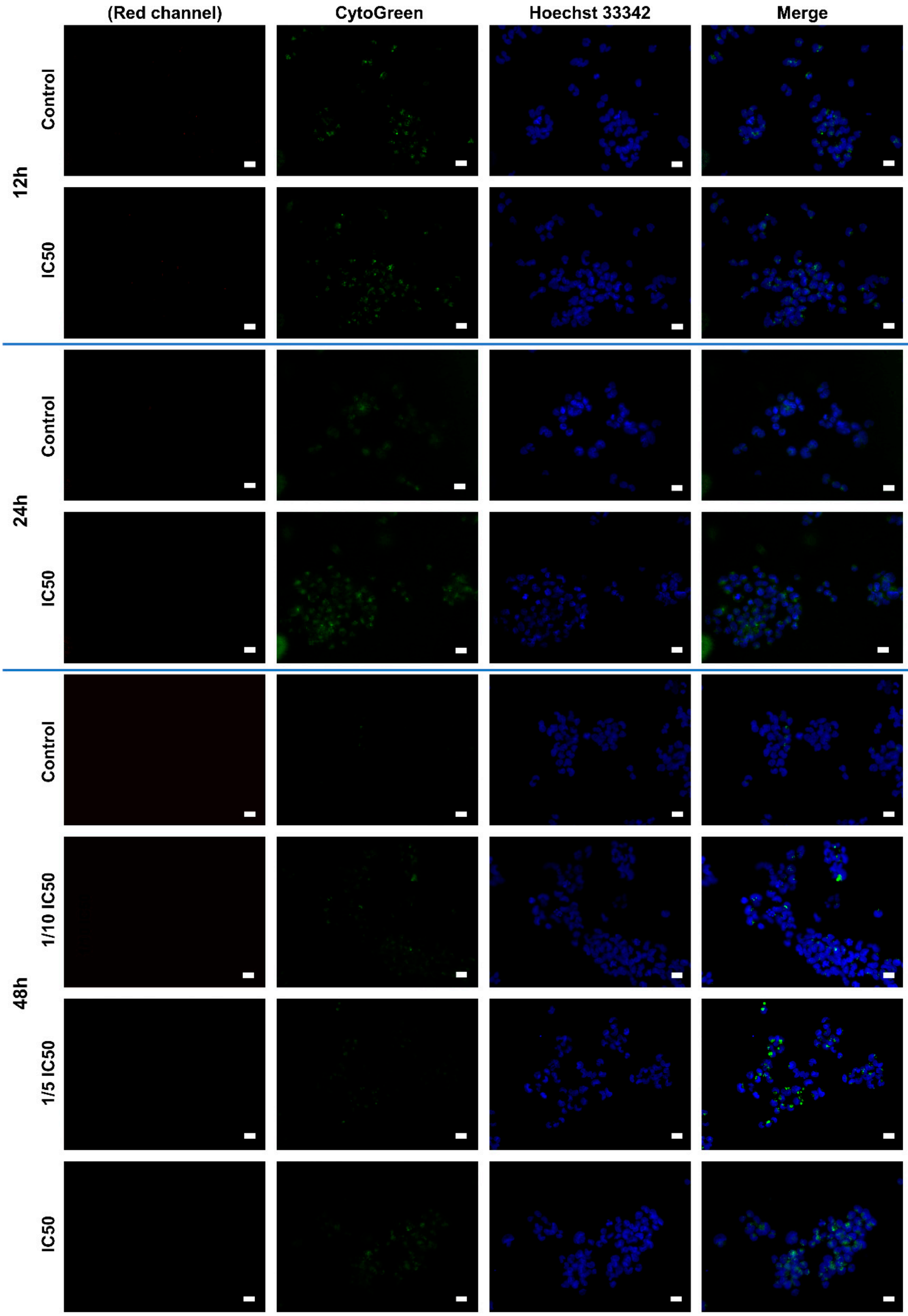

Figure S5. Autophagy assay of cells exposed to the extract and the control (PBS) during 12 h, 24 h and 48 h to an extract concentration of IC_{50} (0.08 μg. μL^{-1}) and during 48 h to a concentration of 1/10 IC_{50} and 1/5 IC_{50}. A2780 cell lines stained with CytoGreen (green) for autophagy and counterstain with hoechst 33342 (blue).

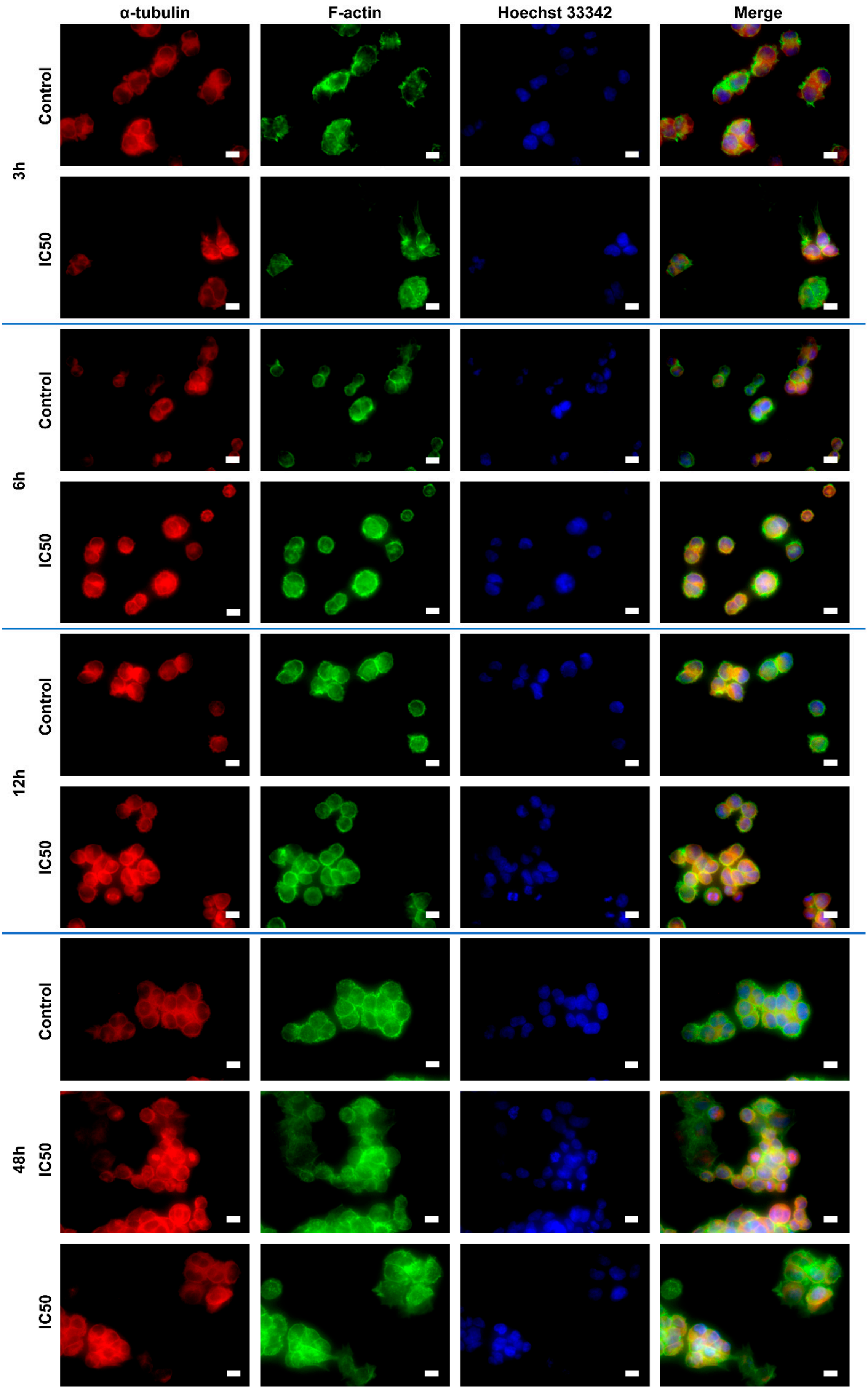
α-tubulin
F-actin
Hoechst 33342
Merge
Control
IC50
3h
Control
IC50
6h
Control
IC50
12h
Control
IC50
48h
IC50

Figure S6. Immunofluorescent assay used in A2780 cells exposed to the extract (IC_{50}, 0.08 μg. μL^{-1}) and the control (PBS) during 3 h, 6 h, 12 h and 48 h. The cells were probed with F-actin (green) and α-tubulin (red) and counterstained with Hoechst dye 33258 (blue).

Table S2. Individual results from STRING for each of the homologous in *Homo sapiens* (ARSB - arylsulfatase B, PRSS1 - trypsin-1 serine protease, YWHAZ - 14-3-3 protein zeta, RAB3C - Ras-related protein Rab-3C, HSPA8 - Heat shock protein 70 kDa), for the proteins identified in *Eulalia* sp.. The network for each protein has been expanded to additional ten proteins and the confidence cut-off for showing interaction links has been set to medium (0.400).

Protein	Biological process				Molecular function				Cellular component				KEGG pathway			
	GO-term	description	count in gene set	false discovery rate	GO-term	description	count in gene set	false discovery rate	GO-term	description	count in gene set	false discovery rate	GO-term	description	count in gene set	false discovery rate
ARSB	GO:0006027	Glycosaminoglycan catabolic process	3 of 62	1.1E-4	GO:0004065	Arylsulfatase activity	2 of 12	0.00018	GO:0005788	Endoplasmic reticulum lumen	4 of 299	3.87E-05	hsa04142	Lysosome	4 of 123	1.48E-07
	GO:1903510	Mucopolysaccharide metabolic process	3 of 109	1.9E-4	GO:0004553	Hydrolase activity, hydrolysing O-glycosyl compounds	2 of 99	0.0033	GO:0043202	Lysosomal lumen	3 of 94	5.49E-05	hsa00531	Glycosamino-glycan degradation	3 of 19	1.48E-07
	GO:0030207	Chondroitin sulphate catabolic process	2 of 14	3.8E-4												
PRSS1	GO:0022617	Extracellular matrix disassembly	6 of 58	1.84E-10	GO:0004252	Serine-type endopeptidase activity	7 of 180	9.11E-11	GO:0031012	Extracellular matrix	5 of 283	1.52E-05	hsa04657	IL-17 signalling pathway	3 of 92	3.90E-04
	GO:0030574	Collagen catabolic process	5 of 37	3.42E-09	GO:0004222	Metalloendo-peptidase activity	5 of 110	2.53E-08	GO:0005576	Extracellular region	9 of 2505	1.52E-05	hsa04926	Relaxin signalling pathway	3 of 130	5.30E-04
YWHAZ	GO:1900740	Positive regulation of protein insertion into mitochondrial membrane	5 of 25	2.45E-09	GO:0019904	Protein domain specific binding	6 of 706	1.20E-04	GO:0005739	Mitochondrion	7 of 1531	4.80E-04	hsa04151	Pl3K-Akt signalling pathway	8 of 348	1.57E-10
	GO:0043065	Positive regulation of apoptotic process	7 of 604	6.17E-07	GO:0019899	Enzyme binding	8 of 2197	2.00E-04	GO:0042470	Melanosome	3 of 105	1.40E-03	hsa04114	Oocyte meiosis	6 of 116	1.05E-09
													hsa04110	Cell cycle	6 of 123	1.05E-09
RAB3C	GO:0015031	protein transport	9 of 1391	6.81E-07	GO:0003924	GTPase activity	9 of 283	1.56E-13	GO:0030659	Cytoplasmic vesicle membrane	7 of 724	1.83E-06	HSA-8873719	Rab geranylgeranylation	9 of 63	7.61E-20
	GO:0016192	vesicle-mediated transport	8 of 1699	1.40E-05	GO:0005525	GTP binding	9 of 366	7.55E-13	GO:0008021	Synaptic vesicle	5 of 155	1.83E-06	HSA-9007101	Rab regulation of trafficking	5 of 118	2.56E-08

HSPA8	GO:1900034	regulation of cellular response to heat	3 of 45	1.40E-04	GO:0051082	Unfolded protein binding	3 of 106	2.80E-04	GO:0000974	Prp19 complex	2 of 12	9.40E-04	hsa04141	Protein processing in endoplasmic reticulum	3 of 161	1.30E-04
	GO:0061684	Chaperone-mediated autophagy	2 of 4	3.00E-04	GO:0051087	Chaperone binding	2 of 100	1.65E-02	GO:0071007	U2-type catalytic step 2 spliceosome	3 of 187	1.20E-03	hsa03040	Spliceosome	3 of 130	1.30E-04
